# Pathogen spectrum identified by targeted nanopore sequencing and exploratory analysis of hematological biomarkers in suspected intracranial infections

**DOI:** 10.3389/fneur.2026.1787441

**Published:** 2026-04-13

**Authors:** Zhou He, Hu Zhihong, Li Shuijin, Jiang Xuyang, Chen Wenhuo, Xu Yongjun, Cui Xiaoping

**Affiliations:** 1 Department of Neurology, Second Department of Infection, 900th Hospital of PLA Joint Logistic Support Force, Fuzhou, Fujian, China; 2 Department of Neurology, 900th Hospital of PLA Joint Logistic Support Force, Fuzhou, Fujian, China; 3 Department of Emergency, 900th Hospital of PLA Joint Logistic Support Force, Fuzhou, Fujian, China; 4 Department of Cerebrovascular Diseases, Fujian Medical University Affiliated Union Hospital, Fuzhou, Fujian, China

**Keywords:** C-reactive protein, diagnosis, hospital stay, neutrophil ratio, targeted nanopore sequencing

## Abstract

**Background:**

Intracranial infections are severe, life-threatening conditions often caused by diverse pathogens. Targeted nanopore sequencing (TNPseq) offers rapid and accurate pathogen identification, addressing the limitations of traditional diagnostic methods. Besides the pathogen types, factors like inflammation levels also affect treatment outcomes. Thus, we combined TNPseq with clinical indicators to characterize pathogen-specific host-response heterogeneity and optimize clinical stratification in infected patients.

**Methods:**

This retrospective study included 255 patients with suspected intracranial infections, among whom 39 with complete clinical and hematological data were analyzed. Pathogen detection was conducted using TNPseq, and the consistency of pathogen distributions between the full and screened cohorts was evaluated. The diagnostic performance of C-reactive protein (CRP), neutrophil ratio (NR), and procalcitonin (PCT) was assessed via logistic regression and receiver operating characteristic (ROC) curve analysis. Mediation analysis was used to explore the relationship between CRP, NR, and the length of hospital stay (LOS).

**Results:**

The most common pathogens identified were *Propionibacterium acnes*, Human herpesvirus 4, and *Moraxella osloensis*, with similar distributions in both cohorts. Among biomarkers, CRP had the highest diagnostic accuracy, followed by PCT and NR. Additionally, NR and CRP were closely related to the LOS. Mediation analysis suggested that NR fully mediated the effect of CRP on LOS, with higher NR levels associated with longer hospital stays.

**Conclusion:**

TNPseq effectively identifies a broad range of pathogens in intracranial infections. CRP and NR serve as critical indicators of inflammatory burden and host-response heterogeneity, aiding in risk stratification and personalized management when integrated with TNPseq.

## 1 Introduction

Intracranial infections are life-threatening disease and often caused by bacterial, viral, fungal or parasitic pathogens (1). Nowadays, intracranial bacterial infection is still the main cause of morbidity and mortality in neurosurgery cases (2). Globally, intracranial infections have a mortality rate ranging from 3 to 33%, with survivors experiencing long-term neurological sequelae, such as hearing loss, cognitive impairment, and motor deficits (3).

The diagnosis of intracranial infections remains challenging due to the non-specific nature of initial symptoms, which may include headache, fever and impaired consciousness (4–6). Despite advances in medical diagnostics and therapeutics, these infections continue to carry high morbidity and mortality rates, especially when diagnosis and treatment are delayed. At present, cerebrospinal fluid (CSF) culture remains the gold standard for diagnosis of intracranial infections (7), but culture methods are time-consuming, low positive rate and not all species can be cultured, which lead to the deterioration of the patient’s condition (8–10).

Targeted nanopore sequencing (TNPseq) is a next-generation sequencing technique that allows for the identification of pathogens at the species level and can simultaneously detect multiple pathogens by long-read length and targeted amplification, including bacteria, fungi, and viruses (11). A large number of studies has demonstrated that, compared with traditional methods such as microbial culture and PCR, nanopore-targeted sequencing has significant advantages in pathogen detection, including shorter turnaround time, and extremely high accuracy (12–14).

In addition, blood markers also shown an essential value in the diagnoses and prognosis of intracranial infections caused by pathogens (14). Elevated levels of some biomarkers, such as C-reactive protein (CRP) and procalcitonin (PCT), are often both associated with bacterial infections and the inflammatory response of the body to infection (15, 16). Further studies have also confirmed that blood biomarkers can effectively predict the prognosis of bacterial infections. For example, Liang et al. found that combining the variations of neutrophil-to-lymphocyte ratio (NLR) and PCT levels provides good predictive value for shock in septic shock (17).

Therefore, in this study, we aimed to use TNPseq for the precise identification of pathogens in patients with intracranial infections. Subsequently, we analyzed the diagnostic and prognostic value of blood biomarkers in these patients to provide a theoretical basis for early and rapid diagnosis and management.

## 2 Method

### 2.1 Study design and patient selection

This retrospective study included 255 patients with suspected intracranial infections who were admitted to a tertiary hospital between October 2022 and May 2024. All patients underwent pathogen detection using TNPseq. The inclusion criteria were: (1) diagnosis consistent with intracranial infection guidelines; (2) age ≥ 18 years; and (3) detailed pathogen detection results and blood indices: (4) pathogens were identified in CSF of patients using TNPseq. Exclusion criteria included: (1) incomplete data; and (2) severe immunodeficiency or recent antibiotic treatment.

These data were collected through the electronic medical record system of 900th Hospital of PLA Joint Logistic Support Force. Due to the retrospective nature of the study, the Ethics Committee of China People’s Liberation Army Joint Logistics Security No.900 Hospital Former General Hospital waived the requirement for informed consent. The study protocol was approved by the Ethics Committee of China People’s Liberation Army Joint Logistics Security No.900 Hospital Former General Hospital (Approval Number.: 2024–042) and conducted in accordance with the principles of the Declaration of Helsinki.

### 2.2 Targeted nanopore sequencing (TNPseq)

According to previous studies, TNP has been proved to be as accurate as traditional bacterial culture in pathogen diagnosis, so it can be directly used as a diagnostic standard (18, 19). CSF samples from each patient were analyzed using TNPseq. At first, DNA was extracted and purified from CSF using QIAamp DNA microbiome kit (QIAGEN, Canada). Then, the quantification of the extracted DNA was performed using Qubit 4.0 Fluorometer (Thermo Fisher Scientific, MA, USA). Subsequently, the extracted DNA underwent PCR amplification of the target regions using the ABI 2720 Thermal Cycler (Applied Biosystems, USA). Following the instructions provided by the Native Barcoding Expansion 1–12 kit (Oxford Nanopore Technologies, UK), the purified PCR products were labeled with barcodes to enable multiplexing. After barcoding, a 1 μL aliquot was taken, and the concentration of the labeled DNA was measured again using the Qubit 4 Fluorometer (Thermo Fisher Scientific, MA, USA). The pooling libraries were prepared using the DNA Ligation Kit SQK-LSK110 (Oxford Nanopore Technologies, UK) according to the protocol of manufacturer. Next, the libraries were pooled and loaded onto an Oxford Nanopore GridION X5 sequencing instrument (Oxford Nanopore Technologies, UK) for real-time sequencing. The entire sequencing process was performed by Hangzhou Shengting Medical Technology Co., Ltd. (Hangzhou, China).

### 2.3 The detection of hematological biomarkers

Blood samples were collected for routine hematological analysis within 24 h of patient admission. A Mindray BC-5390 hematology analyzer (Mindray Bio-Medical Electronics Co., Ltd., Shenzhen, China) and corresponding diagnostic kits were used to measure routine blood cell counts and CRP levels. WBC and NLR were calculated based on the routine blood cell count. Serum PCT levels were measured using the Elecsys BRAHMS PCT assay kit (Roche Diagnostics GmbH, Mannheim, Germany) on a Roche Cobas e601 automated analyzer through electrochemiluminescence immunoassay. All procedures were strictly performed in accordance with the instructions provided by the instrument and reagent manufacturers.

### 2.4 Receiver operating characteristic (ROC) curve

The diagnostic value of hematological biomarker was evaluated using univariate logistic regression, line regression and ROC curve analysis. Logistic and line regression was performed using R package survival and rms. ROC curves were generated using the R package pROC, and the area under the curve (AUC) was calculated to quantify the diagnostic performance of each biomarker. AUC values closer to 1.0 indicated better diagnostic performance. All results were visualized using R package ggplot2.

### 2.5 Mediation analysis

To explore the potential mechanisms underlying the association between hematological biomarkers and clinical outcomes, mediation analysis was performed. The mediation model examined the role of the neutrophil ratio (NR) as a mediator in the relationship between CRP levels and the length of hospital stay (LOS). The analysis was performed using the “mediation” R package, with estimates of direct and indirect effects calculated. Additionally, the potential mediating role of other biomarkers, including lymphocyte ratio (LR), was assessed to understand their contribution to the inflammatory response and immune regulation.

### 2.6 Statistical methods

All statistical analyses were performed using R software (version 4.4.1) and SPSS (version 25). Continuous variables were presented as medians and inter-quartile ranges (IQRs), with group comparisons performed using the Mann–Whitney U test. Categorical variables were expressed as frequencies and percentages, and comparisons were made using the chi-squared test. Besides, *p*-value < 0.05 was considered statistically significant.

## 3 Results

### 3.1 The variation and contents of pathogen in patients with intracranial infections

**Figure 1:**
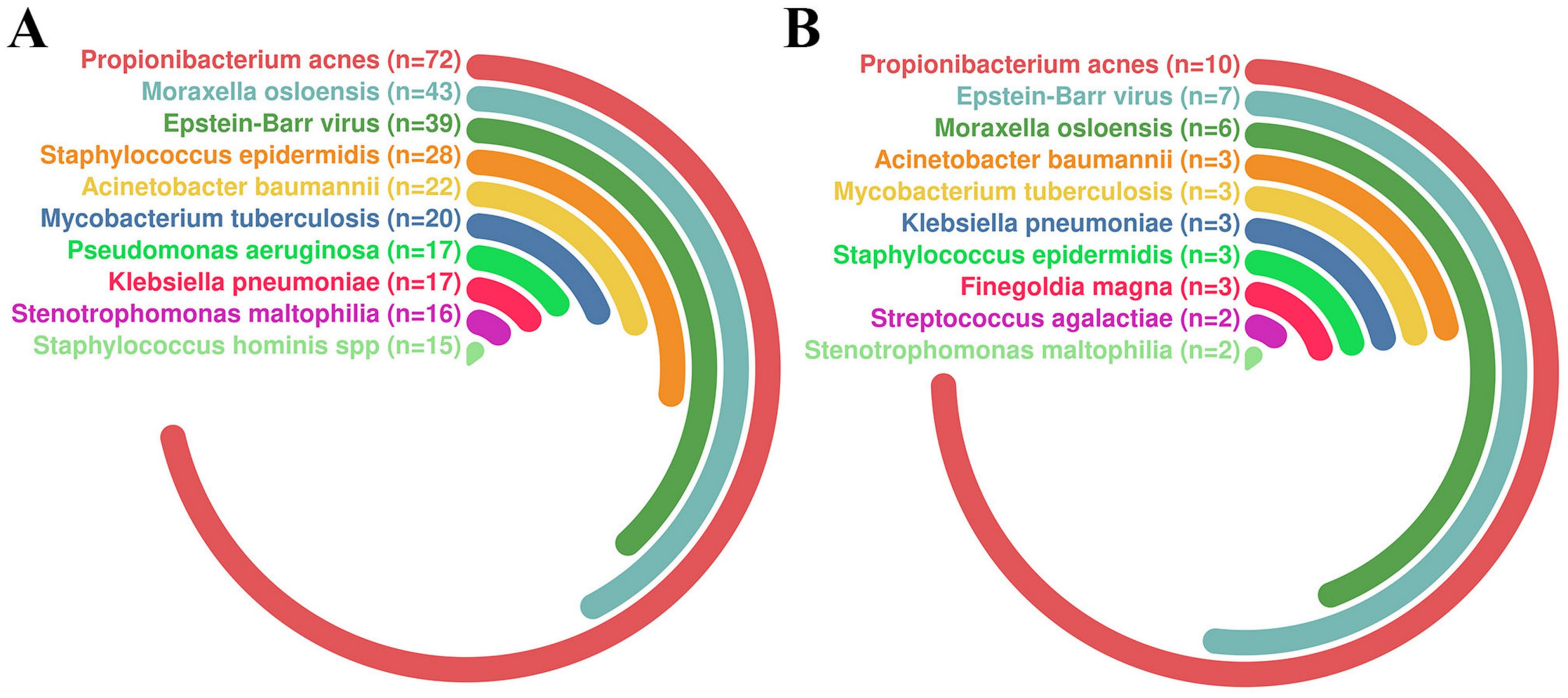
Distribution of Pathogenic Microorganisms Among Patients. **(A)** Distribution of pathogenic microorganisms in the initial cohort of all patients. **(B)** Distribution of pathogenic microorganisms in the screened sample cohort.

To investigate the spectrum of pathogens in patients with intracranial infections, we first analyzed 255 patients using TNPseq technology. The results revealed a diversity of pathogens, with the most commonly detected being *Propionibacterium acnes*, *Moraxella osloensis*, and Human herpesvirus 4 (HHV-4), and were identified in 72, 43, and 39 patients, respectively (Figure 1A).

After excluding samples with missing blood routine examination data, a total of 39 patients meeting the inclusion criteria were reanalyzed (Figure 1B). Although the sample size decreased after screening, the distribution characteristics of the main pathogens remained highly consistent with those in the initial analysis. Notably, *Propionibacterium acnes*, Human herpesvirus 4, and *Moraxella osloensis* continued to rank as the top three pathogens. These findings suggest that the screening process did not significantly alter the overall distribution trends of the major pathogens.

### 3.2 Baseline characteristics of patients with intracranial infections

Among the three most common pathogens, two were bacteria and one was a virus. Considering the differences in the mechanisms of bacterial and viral infections, patients infected with *Propionibacterium acnes* or *Moraxella osloensis* were defined as Group I, while other infected patients were classified as Group II.

Next, we conducted a baseline characteristics analysis of the 39 patients included in the final cohort. A comparison of clinical indicators between Group I and Group II showed no significant differences in age, gender, or antibiotic use. However, several hematological indicators differed significantly between the two groups (Table 1). Specifically, PCT, NR, and CRP levels were significantly lower in Group I than in Group II (*p* < 0.05). Additionally, while the LOS was slightly longer in Group I compared to Group II (19 days vs. 16 days), the difference did not reach statistical significance.

**Table 1:** Baseline characteristics of patients.

Variable	Overall, *N* = 39	Group I, *N* = 13	Group II, *N* = 26	*p*-value
Age, Median (IQR)	50.000 (31.000–63.500)	57.000 (33.000–63.000)	48.000 (29.500–63.750)	0.502
Sex, *n* (%)				>0.999
Female	11 (28%)	4 (31%)	7 (27%)	
Male	28 (72%)	9 (69%)	19 (73%)	
Use of antibacterial, *n* (%)				0.819
No	22 (56)	7 (54)	15 (58)	
Yes	17 (44)	6 (46)	11 (42)	
PCT, Median (IQR)	0.229 (0.143–0.423)	0.100 (0.082–0.166)	0.328 (0.203–0.565)	<0.001
WBC, Median (IQR)	9.330 (6.400–14.465)	10.760 (6.760–14.430)	9.215 (5.165–13.943)	0.484
CRP, Median (IQR)	22.390 (5.351–53.000)	5.600 (3.300–14.900)	42.003 (13.408–74.655)	0.001
NR, Median (IQR)	17.100 (10.565–71.800)	11.680 (5.830–15.340)	46.550 (15.143–74.725)	0.019
LR, Median (IQR)	1.636 (0.765–8.755)	0.890 (0.810–2.290)	3.005 (0.748–12.100)	0.142
LOS, Median (IQR)	16.000 (13.000–20.000)	19.000 (14.000–24.000)	16.000 (13.000–18.000)	0.091

PCT, procalcitonin; WBC, white cell count; CRP, C reactive protein; NR, Neutrophil ratio; LR, Lymphocyte ratio; LOS, length of stay.

### 3.3 Univariate logistic regression and ROC analysis

To further investigate the predictive value of key hematological indicators for diagnosing infections, we performed univariate logistic regression analysis on the three blood indices that showed significant differences. As shown in Table 2, the results indicated that NR, CRP, and PCT were all statistically significant predictors of infection (*p* < 0.05). Among them, CRP demonstrated the strongest association with infection (*p* < 0.05).

**Table 2:** The univariate logistic analysis for the diagnosis of infection.

Name	*N*	OR	95%CI	*p*-value	Adjusted OR	Adjusted 95%CI	Adjusted *p*-value
PCT	39	0.000	[0.0, 0.002]	0.007	0.000	[0.000, 0.001]	0.012
CRP	39	0.936	[0.886, 0.989]	0.020	0.924	[0.865, 0.988]	0.021
NR	39	0.971	[0.945, 0.998]	0.038	0.971	[0.945, 0.999]	0.040

PCT, procalcitonin; CRP, C reactive protein; NR, Neutrophil ratio.

**Figure 2:**
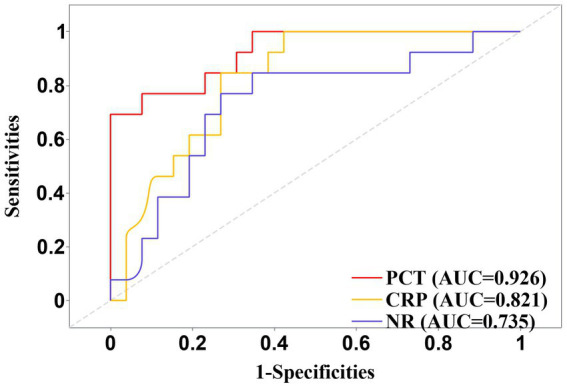
Receiver operating characteristic (ROC) curves of hematological biomarkers for differentiating group I from group II in the screened cohort.

Additionally, ROC curve analysis further validated the diagnostic capability of CRP, with an AUC value of 0.812, indicating high diagnostic efficiency (Figure 2). The AUC values for PCT and NR were 0.768 and 0.754, respectively, also demonstrating good diagnostic value.

### 3.4 Linear regression analysis

**Figure 3:**
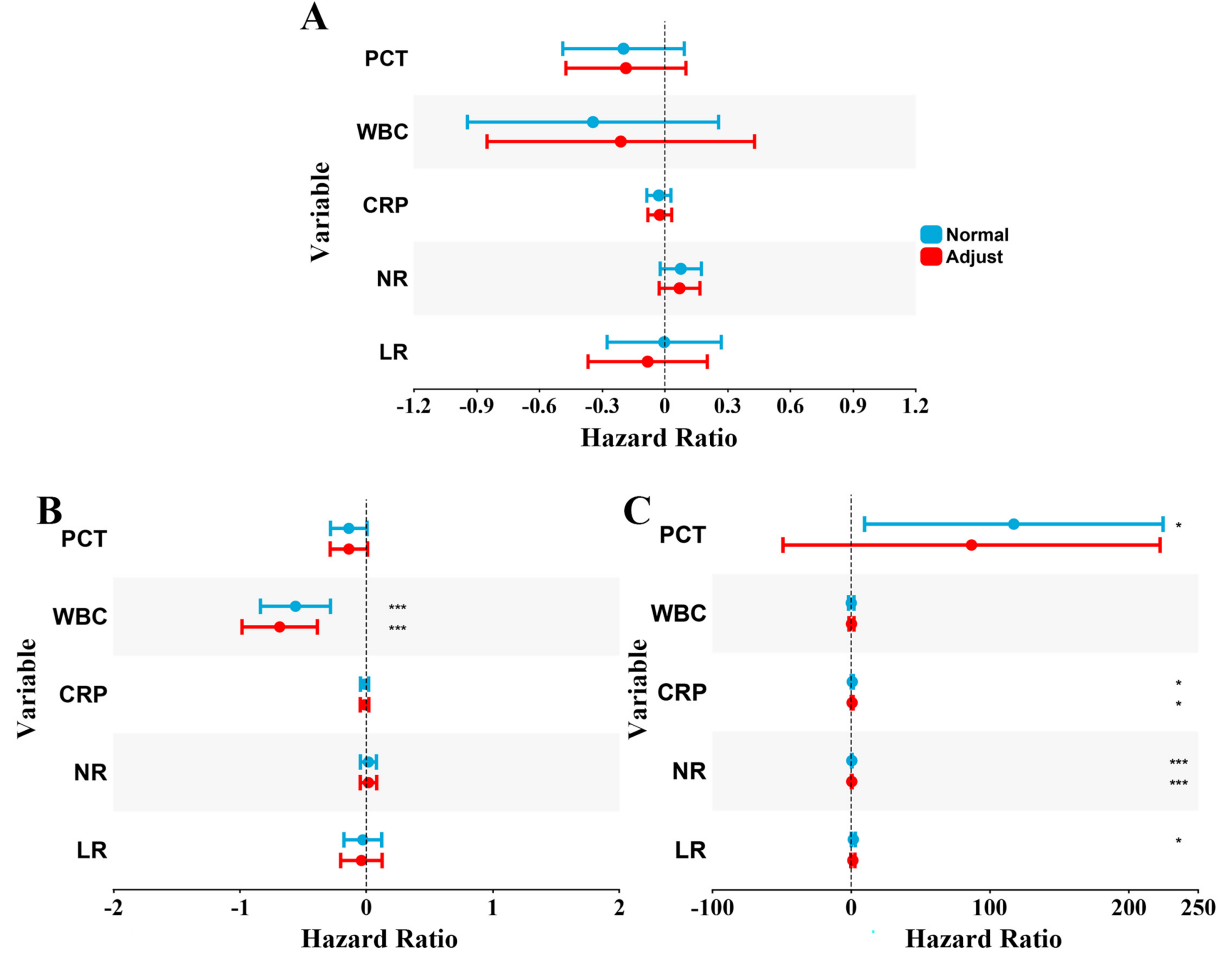
Forest plots of linear regression analyses evaluating the association between hematological biomarkers and length of hospital stay (LOS). **(A)** The linear regression analysis for all patients. **(B)** The linear regression analysis for Group II. **(C)** The linear regression analysis for Group I. Each panel displays the results of multivariable linear regression. Points represent the standardized regression coefficients, which indicate the direction and magnitude of the association between the biomarker and LOS. Horizontal lines represent the 95% confidence intervals (CI). A point to the left of the vertical dashed line (zero effect) indicates a negative association (higher marker level linked to shorter LOS), while a point to the right indicates a positive association.

In addition to diagnostic value, we also tried to evaluate the impact of hematological indicators on the LOS, so we performed a linear regression analysis, adjusting for variables including antibiotic use (Figure 3A). However, in the uninfected group, lower WBC levels were significantly associated with longer hospital stays (Figure 3B). In Group I, higher levels of PCT, CRP, NR, and LR were significantly associated with shorter hospital stays (Figure 3C). Notably, when the use of antibiotic was included as an adjustment variable, PCT and LR did not show significant predictive value for LOS in Group I. Given the limited sample size and subgroup fragmentation, this finding should be interpreted with caution. Notably, these associations were no longer statistically significant after adjustment for antibiotic use, suggesting potential confounding by treatment-related factors.

### 3.5 Correlation analysis

**Figure 4:**
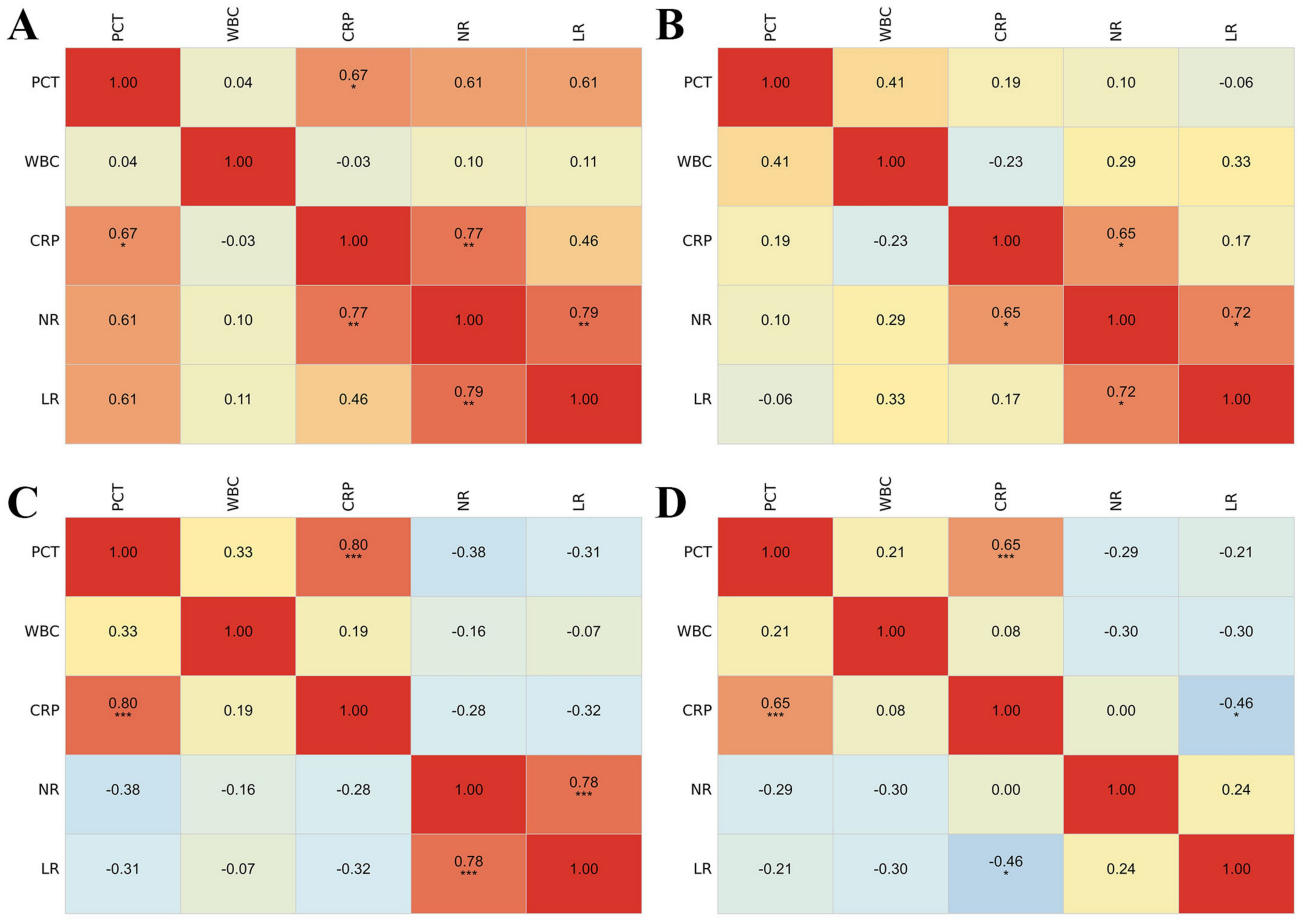
Correlation heatmaps of hematological biomarkers stratified by subgroup and antibiotic exposure. **(A)** Correlation heatmap for the Group I with the use of antibiotic. **(B)** Correlation heatmap for the Group I without the use of antibiotic. **(C)** Correlation heatmap for the Group II with the use of antibiotic. **(D)** Correlation heatmap for the Group II without the use of antibiotic. Correlation matrices are shown for different subgroups based on infection status and antibiotic use. The color gradient from blue to red represents the Pearson correlation coefficient, ranging from −1.0 (perfect negative correlation) to 1.0 (perfect positive correlation).

Then, to further explore the interactions among hematological indicators, we conducted correlation analysis. The results showed significant differences in the correlations of hematological indicators between the infection and Group II (Figure 4). In the antibiotic-treated Group I, there were significant positive correlations between CRP and PCT, NR and CRP, and LR and NR (Figure 4A). However, in Group I, among those who did not receive antibiotics, the correlation between CRP and PCT was not observed (Figure 4B). In antibiotic-treated patients in Group II, significant positive correlations were found between CRP and PCT, and between LR and NR (Figure 4C). In contrast, patients who did not receive antibiotics, the significant correlation between LR and NR was not observed, but a significant negative correlation between LR and CRP was identified (Figure 4D).

### 3.6 Mediation analysis

**Figure 5:**
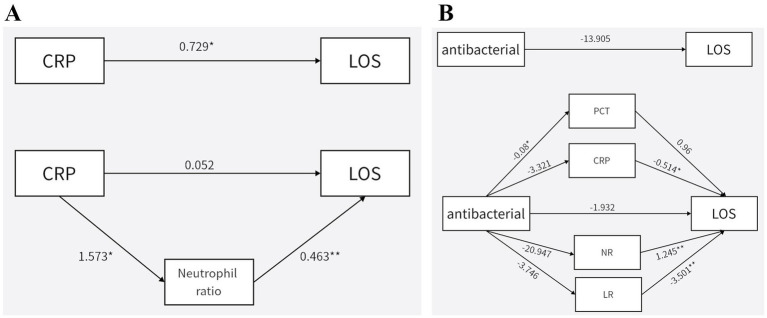
Exploratory mediation analyses of biomarker associations with length of hospital stay. **(A)** The association between CRP and LOS based on mediation analysis. **(B)** The association between antibacterial and LOS based on mediation analysis. The paths labeled ‘a’, ‘b’, and ‘c’ represent regression coefficients between variables.

Finally, to preliminarily explore its mechanism of hematological indicators, the mediation analysis was performed. The results of mediation analysis revealed that the effect of CRP on the LOS in Group I was fully mediated by NR, suggesting the central role of NR in the inflammatory response to infection (Figure 5A). Additionally, the impact of antibiotic use on LOS was entirely dependent on LR, emphasizing the importance of LR in immune regulation (Figure 5B).

## 4 Discussion

In this study, we employed TNPseq to investigate the pathogen spectrum in intracranial infections and explored the diagnostic and prognostic value of key hematological biomarkers. Our findings reveal novel insights into the relationship between pathogen distribution, inflammatory markers, and clinical outcomes, contributing to the understanding and management of intracranial infections. At first, *Propionibacterium acnes*, Human herpesvirus 4, and *Moraxella osloensis* were identified as the most common pathogens according to the results of TNPseq. In addition, we identified dozens of other pathogenic microorganisms, including various bacteria, fungi, and viruses (data not shown). Similarly, a study also indicated that *Propionibacterium acnes* is one of the major pathogens in central nervous system infections (20). However, Ruan et al. identified Acinetobacter and Staphylococcus as the primary pathogens in patients with intracranial infections using high-throughput sequencing technology (5). Additionally, a meta-analysis found that the incidence of *Acinetobacter baumannii*-related intracranial infections has been increasing in recent years (21). These findings further highlight the diversity of pathogens causing intracranial infections. Although our results differ from these studies, this discrepancy may be due to various factors, such as geographic differences and detection methods. Furthermore, intracranial infections can present with diverse clinical manifestations, and different pathogens may cause different clinical symptoms. Therefore, the variations in clinical presentations among patients included in different studies may contribute to the inconsistency between our findings and previous research.

A great deal of studies has shown that PCT and CRP can effectively predict bacterial infections (22–24). Li et al. found that PCT and high-sensitivity CRP (hsCRP) levels were significantly lower in children with bacterial infections compared to the non-infection group (25). More importantly, this study also demonstrated that PCT and hsCRP had the best predictive performance for bacterial infections in children (25). Norman-Bruce et al. confirmed that for invasive bacterial infections, the AUC value of PCT was higher than that of CRP (22). Additionally, the NR has also been recognized for its diagnostic value in bacterial infections. A retrospective study indicated that in emergency care settings, a lower NLR might offer better predictive value than CRP levels (26). Similarly, we found that PCT, CRP, and NR had good diagnostic value for bacterial infections, with the AUC of PCT being higher than that of CRP and NR. These findings suggested that the accuracy of blood biomarkers in bacterial infections may be influenced by factors such as the type of infection. Therefore, in clinical diagnosis, it is necessary to make comprehensive judgments based on multiple indicators rather than excessively relying on one indicator.

CRP is one of the typical markers of inflammation and infection (27). Many studies have shown that CRP levels significantly increased after bacterial infections (28). However, Shen et al. found that the extent of CRP elevation varied depending on the infecting pathogen (29). On the other hand, neutrophils are critical immune cells involved in bacterial clearance, and patients with neutropenia are more prone to severe bacterial infections (30). In most studies about bacterial infections, neutrophil levels tended to increase (31–33). However, some bacterial infections could lead to reduced neutrophil counts. For example, Shigella can suppress neutrophil recruitment by downregulating ICAM-1 (34). These findings suggested that bacteria have heterogeneous effects on immune responses. Similarly, our results showed that in patients infected with *Propionibacterium acnes* or *Moraxella osloensis*, CRP and NR levels were significantly lower than in patients infected with other bacteria. It suggested that host inflammatory responses may differ across pathogen-defined subgroups.

Further regression and mediation analyses showed an association between CRP, NR, and LOS in Group I. Studies have demonstrated that CRP can modulate inflammation and neutrophil recruitment either positively or negatively by inhibiting neutrophil chemotaxis or upregulating adhesion molecules and pro-inflammatory cytokines (35, 36). Neutrophils have also been shown to play dual roles in infections (37). Except eliminating pathogens, neutrophil recruitment to the brain may cause tissue damage and exacerbate neurological injuries (38). Consequently, neutrophils have become recognized as key drivers of hyperinflammatory responses leading to mortality in bacterial infections (39). However, the inverse association between inflammatory biomarkers and LOS observed in Group I was observed. Previous studies demonstrated that higher biomarker levels at presentation may trigger earlier clinical recognition and more timely therapeutic intervention, including antibiotic administration, which could shorten hospital stay (40, 41). Importantly, the loss of statistical significance after adjustment for antibiotic use suggests that treatment-related factors, such as the duration and complexity of the antimicrobial regimen, play a substantial role in determining the total length of stay (42).

In conclusion, this study describes the pathogen spectrum identified by TNPseq and the exploratory associations of hematological biomarkers in suspected intracranial infections. The integration of TNPseq with biomarker analysis offers a multi-dimensional assessment framework, enabling both precise pathogen identification and accurate stratification of patients based on inflammatory intensity.

However, this study has several limitations. The relatively small sample size of the screened cohort limits the generalizability of the findings, which need to be validated in a large cohort. Additionally, our results are inferred from existing results and known literature to some extent, and need to be verified by *in vivo*, *in vitro* or clinical experiments.

## Data Availability

The raw data supporting the conclusions of this article will be made available by the authors, without undue reservation.
